# Evidence of Inter-Professional and Multi-Professional Interventions for Geriatric Patients: A Systematic Review

**DOI:** 10.5334/ijic.4683

**Published:** 2020-02-24

**Authors:** Elisabeth Platzer, Katrin Singler, Peter Dovjak, Gerhard Wirnsberger, Annemarie Perl, Sonja Lindner, Aaron Liew, Regina Elisabeth Roller-Wirnsberger

**Affiliations:** 1 Department of Internal Medicine, Medical University of Graz, Graz, AT; 2 Institute for Biomedicine of Ageing, Friedrich-Alexander University Erlangen-Nürnberg, Nürnberg, DE; 3 Department of Geriatrics, Klinikum Nürnberg, Paracelsus Private Medical University, Nürnberg, DE; 4 Salzkammergutklinikum Gmunden, Department of Acute Geriatrics, Gmunden, AT; 5 Austrian Society on Geriatrics and Gerontology, Vienna, AT; 6 Portiuncula University Hospital, Ballinasloe, IE; 7 Clinical Sciences Institute, National University of Ireland, Galway, IE

**Keywords:** multiprofessional, integrated care, setting, evidence, systematic review

## Abstract

The current demographic shift raises the demand for provision of health care tailored to the complex care needs for older adults. Given the growing number of national care plans and best practice models there is an urgent need to build evidence for inter- and multiprofessional care provision for older people when offered an integrated care approach.

The aim of this study was to determine whether an inter-professional or multi-professional care intervention, can improve geriatric patients’ health determinants.

A systematic review was performed according to PRISMA Guidelines. Databases were searched for clinical trials which compare inter-professional or multi-professional complex care interventions with usual care among people aged ≥60 years, in hospital or emergency care settings.

Based on nine studies, inter-professional or multi-professional intervention has no impact on mortality rate but either positive or neutral effects on physical health, psychosocial wellbeing and utilization of health care service. It shows that these inter-professional or multi-professional interventions were feasible.

This systematic review highlights the scarcity of evidence showing either positive or neutral impact of intervention based on inter-professional or multi-professional teamwork across care settings on the health determinants among geriatric patients. International harmonization of assessment tools may allow direct comparisons for future interventions.

## Background

The global utilization of health care system by older adults is increasing, parallel with an aging population [1,2,3]. Aging is often associated with co-morbidity and impaired functional reserve, necessitating personalized and comprehensive medical care [4,5,6].

The European Health Programme highlights the role of integrated care with the specific aims to improve patient experience, outcomes of care and effectiveness of health systems (known as “triple aim”) through linkage or coordination of services and providers along the continuum of care [7]. In parallel, the World Health Organization emphasizes on the role of effective and sustainable collaborative network among professionals across all medical care settings, with the aims to improve health outcomes and reduce healthcare cost [8,9,10]. However, the exact nature of these interactions among different professions on health outcome and overall cost, is currently unclear [9,11]. Furthermore, robust evidence on the efficacy of complex care interventions based on multi-professional teams and integrated interventions, remains scarce.

Therefore, the aims of this systematic review are two folds. First, we sought to evaluate the impact of intervention based on multi-professional teamwork across care settings on the health determinants among geriatric patients. Secondly, we sought to determine the specific profession(s) which could lead to sustainable benefits for patient and health care systems.

## Method

This systematic review was conducted according to PRISMA guidelines and was registered in PROSPERO (CRD42018097024).

### Data resources and search strategies

Relevant clinical trials published between 1^st^ Jan 2008 and 31^st^ December 2018 in English or German languages were identified using PubMed, Cochrane, CENTRAL, CINAHL, Medline and Embase database. Search strategy using the following Medical Subject Headings: “treatment outcome”, AND “aged” AND “Patient Care Team”. If required, the Medical Subject Headings were adapted to the specific database options with synonyms of the Medical Subject Headings. Further search via greylit.org and reference tracking were performed to identify additional studies.

### Inclusion Criteria

To be included, trials must meet all of the following criteria: (1) Randomized controlled trials or non-randomized controlled trials; (2) compares inter-professional or multi-professional complex care interventions with usual care; (3) included people aged 60 years or older; and (4) admission to a hospital or emergency care setting. The intervention must demonstrate an integrated care approach by various professionals from the hospital or emergency care setting, with outreach to difference care settings. Inter-professional interventions link between disciplines into a coordinated and coherent whole. Multi-professional interventions are based on knowledge of different disciplines but stays in the boundaries of these fields [12].

### Outcomes of interest

The primary outcome were physical health, psychosocial wellbeing, mortality, and utilization of health care services including length of stay, and admission and readmission rates in the hospital setting. The secondary outcome was the exact composition of the inter-professional and multi-professional teams. The results were sub-categorized into micro, meso and macro levels [13].

### Quality assessment

The risk of bias of the included studies were assessed by two independent reviewers (E.P. and R. E. R-W.) using the Critical Appraisal for Therapy Articles Worksheet – Centre for Evidence-based Medicine, University of Oxford 2005 [14]. The independence of all reviewers was ensured by local separation. After evaluation, results were compared and discussed until a consensus was reached. Disagreement was resolved by a third independent reviewer (K.S or P.D.).

### Data synthesis and analysis

Meta-analysis was not performed due to the expected heterogeneity of the interventions. Relevant outcome data from the included studies will be summarized and appraised.

## Results

**Figure 1:**
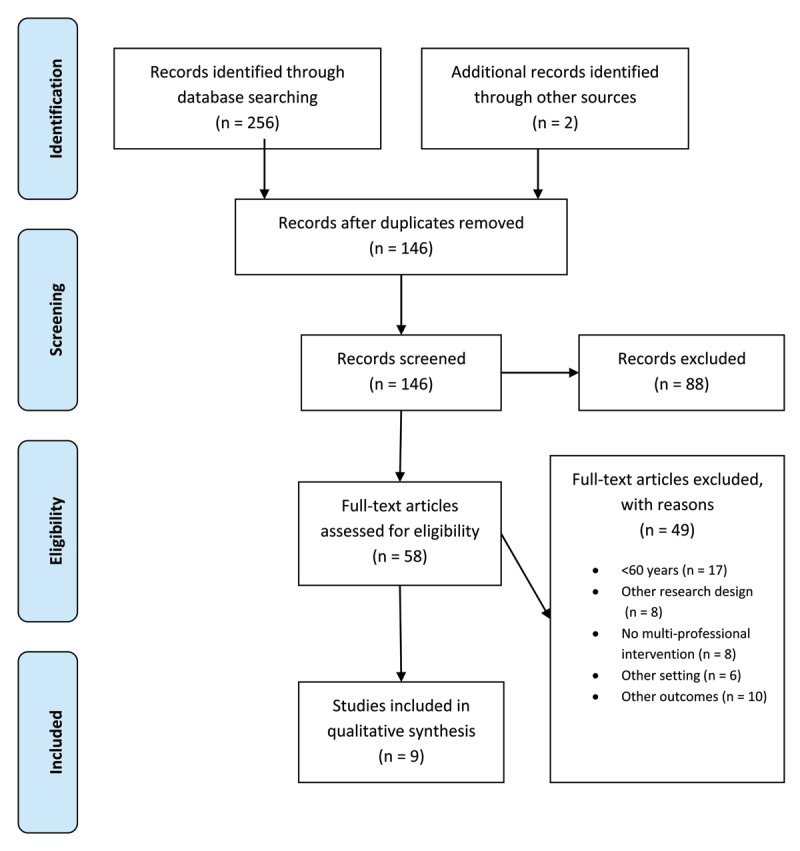
Flow chart. The flowchart illustrates the search strategy applied to answer the research question outlined. In total 258 studies were identified during the systematic data search (256 in scientific literature, two additional publications by hands-on search). Following qualitative evaluation and screening full text, only nine studies fulfilled predefined inclusion criteria of the study and were further processed in the review process.

A total of 256 relevant citations were identified through search strategy. Two additional studies were detected by hands-on search. After cleaning from duplicates, one reviewer screened titles and abstracts to exclude papers that were clearly not relevant to the research question. After that, a total of 58 full-text studies were assessed for eligibility. Finally, nine studies, involving a total of 1,739 participants, met the inclusion criteria. The PRISMA diagram illustrates the selection process of the studies and shows reasons for exclusion (Figure 1).

### Results from the quality assessment

Table 1 demonstrates the results of the quality assessment run for the studies finally included into the systematic review. As may be seen there was homogeneity between the studies concerning the quality of randomization and group characteristics between the intervention groups. Substantial inconsistency was found for description of other treatments and interventions offered to participants during the inter- or multiprofessional complex care, also affecting the outcomes addressed.

**Table 1:** Summary of the risk of bias using Critical Appraisal for Therapy Articles Worksheet [14].

Oxford Critical Appraisal	Azad et al. 2008	Beck et al. 2015	Courntey et al. 2009	Deschodt et al. 2011	Gillespie et al. 2009	Hendriks et al. 2008	Shyu et al. 2010	Shyu et al. 2013	Trombetti et al. 2013

**Selectionbias**									
Was the assignment of patients to treatments randomised?	Yes	Yes	Yes	Yes	yes	yes	yes	Yes	no
Were the groups similar at the start of the trial?	Yes	Yes	yes	Yes	yes	yes	yes	Yes	yes
**Performancebias**									
Aside from the allocated treatment, were groups treated equally?	Yes	Yes	no	yes	yes	yes	unclear	unclear	yes
**Attritionbias**									
Were all patients who entered the trial accounted for? Were they analysed in the groups to which they were randomised?	Yes	Yes	yes	yes	unclear	yes	yes	Yes	unclear
**Observerbias**									
Were measures objective or were the patients and clinicians kept “blind” to which treatment was being received?	No	No	yes	no	no	no	yes	no	yes

The critical appraisal was performed with the Critical Appraisal for Therapy Articles Worksheet – Centre for Evidence-based Medicine, University of Oxford 2005. Possible answers were “yes”, “no” and “unclear”.

### Study characteristics

The baseline characteristics of the nine studies, included in this systematic review were summarized in Table 2. All studies are randomized controlled trials (apart from the study by Trombetti et al.) and were published between 2008 [15] and 2015 [16]. Sample size ranged from 71 [16] to 368 participants [17]. The mean age ranged from 74,9 [18] to 86,8 years [17].

**Table 2:** Study characteristics.

	Patients (n)	Intervention (n)	Comparison (n)	Mean age (years)	Country

Azad et al. 2008	91*	45	46	75,0	Canada
Beck et al. 2015	71	34	37	85,0	Denmark
Courntey et al. 2009	122	58	64	78,8	Australia
Deschodt et al. 2011	171	94	77	80,8	Belgium
Gillespie et al. 2009	368	182	186	86,75	Sweden
Hendriks et al. 2008	333	166	167	74,85	Netherlands
Shyu et al. 2010	162	80	82	78,15	Taiwan
Shyu et al. 2013	299	CC (99) IC (101)	99	76,51	Taiwan
Trombetti et al. 2013	122	92	30	84	Switzerland

Abbreviations: CC= Comprehensive Care, IC= Interdisciplinary Care; * women only.

Seven studies focused on multi-professional studies while only two assessed the effect of inter-professional interventions. The duration of the interventions ranged from the total hospital stay to six months after discharge. Eight studies compared their interventions with usual care group [15,17,18,19,20,21,22,23].

### Nature of intervention

As expected, there was a significant heterogeneity in the nature of intervention (Table 4). Overall, six studies included home based intervention in addition to those within the clinical settings [15,16,18,19,21,22].

### Outcome of inter-professional and multi-professional interventions

#### Effectiveness of interventions on microlevel

##### Physical health

The analysis of the physical functioning by activities of daily living (ADL) was based on five trials using either Barthel index, the 6-item Katz Index, Groningen Activity Restriction Scale or the Chinese Barthel Index as primary endpoints [16,18,20,21,22]. Three studies showed significant improvement in functional status [20,21,22]. Shyu et al. (2010) and Shyu et al. (2013) showed significant improvement in Chinese Barthel Index [21,22]. Deschodt et al. (2011) showed a significant improvement in ADL status within the eight days post-operatively, which was not sustained at one year [20]. In contrast, two other studies showed no significant improvement in ADL [16,18].

Shyu et al (2010) showed that inter-professional intervention led to a significant reduction in falls [22]. However, three other studies showed no effect on the number of falls [18,21,23]. Three studies showed some benefit of multi-professional intervention on either mobility score, handgrip, gait speed maximum, timed up and go, and the walking ability [16,22,23].

##### Mortality

Seven studies evaluated the impact of an intervention on individual mortality, but none of the studies could demonstrate significant reduction of mortality rates due to an inter- or multi-professional intervention [15,16,17,20,21,22,23].

##### Psychosocial well being

Four studies assessed outcomes related to psychological health including quality of life, mental health and depressive symptoms [16,18,21,22].

Shyu et al (2010) showed that inter-professional intervention improved quality of life based on the SF36 score [22]. In contrast, two other studies showed no significant improvement in quality of life [16,18].

Shyu et al. (2013) showed that both the inter-professional and comprehensive interventions significantly lower the risk of depression after one year [21]. Similarly, Shyu et al. (2010) showed that intervention significantly reduces depressive symptoms [22]. In contrast, Hendriks et al (2008) showed that multi-professional intervention has no significant impact on mental health and Depression score [18].

#### Effectiveness of interventions on Meso-level and Macro-level

##### Utilization of health care service

Six studies assessed the re-admission rates to hospital as an outcome on the macrolevel [15,16,17,19,20,22]. The multi-professional liaison team intervention with additional dietitian counselling and home care resulted in a significantly lower re-admission within six month [16]. Similarly, Courtney et al. (2009) showed that intervention significantly reduces readmission rates [19]. On the other hand, Azad et al. (2008) showed a significantly fewer re-admissions, but only for those with chronic heart failure [15]. Three other studies showed no significant effect of intervention on re-admission rates [17,20,22].

Two studies showed that multi-professional intervention has no significant impact on the length of stay in hospital [15,23]. Two studies showed a significant reduction in emergency department visit following multi-professional intervention [15,19].

### Professionals involved in the interventions

Multi-professional and inter-professional team composition of the included studies were summarised in Table 3. All included studies described multi-professional team structures in their interventions. All interventions described physicians and nurses as part of the team [15,16,17,18,19,20,21,22,23]. Four provided service from trained professions with specific experience in geriatric care [16,18,19,20]. Seven studies involved physiotherapists alongside medical and nursing care for falls prevention [13,14,17,18,19,20,21].

**Table 3:** Multi- and inter-professional team composition.

	Physician	Nurse	Physio-therapist	Dietician	Occupational-therapist	Pharmacist	Psychiatrist	Social-worker	Additional partners of care	Inter-disciplinary	Multi-disciplinary

Azad et al. 2008	X	X	X	X	X	X		X	X		X
Beck et al. 2014	(x)	X	X	X	X						X
Courtney et al. 2009		X	X								X
Deschodt et al. 2011	X*	X*	X*		X*			X*			X
Gillespie et al. 2009	X					X					X
Hendriks et al. 2008	X*	X*			X						X
Shyu et al. 2010	X*	X*	X							X	
Shyu et al. 2013	X*	X*	X	X			(X)			X	
Trombetti et al. 2012	X	X	X	X	X	(X)		X			X

* With expertise in geriatric care, (X) can be consulted if necessary.

**Table 4:** Multi-professional and inter-professional interventions and strategies.

Clinical setting					

Author	Design	Strategy	Components of the intervention	CG	Frequency

Deschodt et al. 2011	RCT	Inpatient Geriatric consultation [24]	CGA from nurse to detect potential problems. In-depth multidisciplinary evaluation of assessed problems. Formal clinical advice and recommendations documented in electronic form and discussed in health care team. In-hospital follow-up to check for new problems and if team’s advice were implemented or needed more clarification.	UC	during hospital stay
Gillespie et al. 2009	RCT	Comprehensive pharmacist intervention	After admission the pharmacist summarized patient’s medication list and conducted an interview to give advices for medication intake. During inpatient stay, the pharmacist performed a comprehensive drug review [25], discussed drug related problems with health care team during ward rounds and give advices to patient’s physician. At Discharge the pharmacist provided medication counselling as a complement to the physicians discharge information. A comprehensive discharge letter was faxed to patients GP. To ensure adequate medication home management and record any changes in medication, the pharmacist contacted patients by telephone 2 months after discharge.	UC	Admission to discharge, 2-month telephone follow-up to ensure home management of medications
Trombetti et al. 2013	CT	Multi-disciplinary multifactorial intervention program	Multidisciplinary comprehensive assessment to define fall and fracture risk factors. Followed by an individually tailored intervention this included targeted rehabilitation therapy (physician, physiotherapist, occupational therapist, dietician, nurse, social worker). Additional physiotherapeutic group sessions, eurhythmics workshops and workshops with an occupational therapist. A systematic battery of tests and multidisciplinary team meetings were performed weekly to review and adopt rehabilitation program. Whenever required, a home visit was undertaken before patient’s discharge to assess environmental hazards and facilitate modifications.	UC	5 weekly group sessions (a 60 min) and 3 to 5 individually tailored sessions of 30–45 min. Home visit when required.
**Clinical setting and home based intervention**					

**Author**	**Design**	**Strategy**	**Components of the intervention**	**CG**	**Frequency**

Azad et al. 2008	RCT	Structured multi-disciplinary pathway	Group and home based exercise program (Physiotherapist), nutrition counselling (dietician), energy and stress management (occupational Therapist), counselling patients & families (social worker), CHF education of patients and caregivers (clinic coordinator).	UC	12 visits over 6 weeks and home based exercise program
Beck et al. 2014	RCT	Multidisciplinary discharge liaison-Team with dietician	Discharge Liaison-Team (nurse, occupational Therapist, physiotherapist) test and install aids, review discharge letter, contact GP if relevant and organise home care.. Additional home visits from a dietician to develop and implement individual care plan.	DL	home visits from a dietician at discharge, and after 3 and 8weeks
Courtney et al. 2009	RCT	Discharge Planning and In-home follow-up Protocol (OHP-DP)	Physical exercise intervention from a physiotherapist included muscle stretching, balance training and walking. A nurse developed a transitional care plan including need for assistance, post discharge treatments, follow-up care, social support, chronic disease and medication management. Nurse and physiotherapist combined their visits when panning, explaining and demonstrating exercise program. 48h after discharge, home visit from the nurse to provide and advice support and ensure that exercise program could be safely undertaken at home. Additional home visits were provided if required. Weekly telephone follow-up calls for 4 weeks, followed by monthly calls for 5 months. Contact nurse was possible from 9am to 5pm on weekdays.	UC	Start within 72h after admission and continued through hospitalization. A home visit from a nurse within 48 hours and telephone follow-up for 6 months
Hendriks et al. 2008	RCT	Multidisciplinary fall-prevention program[26]	Structured medical assessment of risk factors for new falls from physician included for example standard examination, vision, sense of hearing, locomotor apparatus, feet and footwear as balance and mobility and the affect (in hospital) Home based assessment from an occupational therapist included functional assessment, environmental hazards and psychological consequences of the fall. Finally a summary of the results were sent to the participant’s GP with recommendations and referrals.	UC	Medical and home based assessment After 2,5–3,5 months all recommendations had to be implemented
Shyu et al. 2010	RCT	Interdisciplinary Intervention for Hip Fracture	Geriatric assessment and consultation from a geriatric nurse and a geriatrician. Inpatient rehab program from physio therapist, geriatric nurse and rehabilitation physician. Continuous rehab included inpatient rehab (nurse, physio therapist, rehab physician) and individual at home rehab program (nurse, physio therapist) At discharge planning a geriatric nurse did predischagre assessment (resources, self-care ability needs, long term care service, and referrals) home environmental modifications. A telephone call was done to remind follow up visits.	UC	2x CGA and Home visits from a geriatric nurse und physio therapist
Shyu et al. 2013	RCT	Interdisciplinary care model and Comprehensive care model	1 interdisciplinary care model: geriatric consultation with medical supervision (nurse and geriatrician), rehab program focused on relieving pain, muscle strength and endurance, discharge planning with post-hospital service (discharge assessment, referrals and reminders for clinical follow-up) 2 Comprehensive care model: included the components of the interdisciplinary care model and additional assessment of nutritional status, depression and fall before discharge. Those with a risk of malnutrition, depression and fall received additional services. The rehab protocol was same for both groups.	UC	Rehab program (4 months in group 1, 6 months in group 2) with home visits from nurse and physio therapist

Abbreviations: CG = control group, RCT = randomised controlled trial, CT = controlled trial, UC = usual care, DL = discharge liaison team, CGA = comprehensive geriatric assessment, CHF = chronic heart failure.

## Discussion

This systematic review highlighted the paucity of evidence on the impact of intervention based on inter-professional or multi-professional teamwork across care settings on the health determinants among geriatric patients. Therefore, the overall results of this systematic review need to be interpreted cautiously. Based on the data from nine studies, the overall evidence remains scarce and inconsistent, most likely, inherent to the nature of the intervention and difference in professionals involved. For instance, one study only included women [13] while the others excluded frail people [20] and those with low renal clearance [17]. One study had a significantly higher mortality rate (32,1%) [15]. The assessment for ADL, quality of life and depressive symptoms differ significantly, rendering direct comparison impossible. Furthermore, the healthcare settings are different worldwide, rendering generalization of the results impossible. But also in similar healthcare systems, the composition for one setting may not be desired or even feasible in another. Even in a practice setting, teams, resources or availability of time may change [27].

Overall, our systematic review showed that inter-professional or multi-professional intervention has no impact on mortality rate, consistent with previous systematic reviews [4,11,28,29]. There are conflicting results, demonstrating either positive or neutral outcome, on physical health, psychosocial wellbeing and utilization of health care service, in contrast with previous systematic reviews [2,29,30].

Our systematic review also demonstrated that inter-professional or multi-professional interventions were feasible. These interventions were performed by various professionals, which were predominantly doctors and nurses [15,16,17,18,19,20,21,22,23]. Apart from objective health outcomes, the questions about composition of teams are of interest in the context of the topic. Some interventions involved physiotherapists, dietitians, occupational therapists and social workers [15,20,21,22,23]. In fact, collaborative interventions by several different professions have been shown to be effective in improving patient-related outcomes [31,32,33]. As could be shown in a recent publication by LaDonna et al. 2017 physicians, nurses as well as pharmacists should be part of the health care team [33]. Similar results were obtained in our review but individuals revealed a broader sense of care team than the traditional definition used by literature. Results of this study indicate that patients identify between paid and unpaid team members as well as housekeepers and spiritual advisors. Therefore, it may be beneficial to ask patients who they consider to be in their team and engage these individuals in collaboration [33]. However, it is not clear in the data from our review which team composition is the most favourable for patients in transition from hospital to other care settings. This important result of the current review highlights the need for a broadly accepted and consented framework of collaboration in inter- and multiprofessional teams.

Currently, the comprehensive Geriatric Assessment (CGA) remains the core element of evidence for integrated complex care management of older patients [6]. The Cochrane Review by Ellis et al. (2017) [4] and other studies [2,11,28], provided evidence for the effectiveness of multi-professional comprehensive geriatric care when extended to different care settings on health care utilization. Our systematic review highlights the importance of international harmonization of assessment tools, especially for physical health and psychosocial wellbeing, within the CGA, to allow direct comparisons for future interventions.

## Limitations

Our systematic review has several limitations. Firstly, there are inconsistencies of several results, inherent to the heterogeneous nature of the intervention and multi-professional involvement. Secondly, despite low risk of overall selection bias, there were some risk for performance bias [19,21,22] and attrition bias [17,23]. There was also a considerable risk for observer bias due to the characteristics of the interventions [15,16,17,18,20,21]. Thirdly, the generalisability of these results may be limited due to the inherent differences in various healthcare systems and the availability of these interventions [27]. Fourthly, this is the most updated systematic review based on an extensive search of studies in both the German and English languages only. Despite this, we found no relevant studies in German language. Finally, published articles were expected to be more likely to report positive findings as compared with unpublished articles. However, we have specifically selected RCTs to mitigate this risk. The work was performed according to best evidence. However, it cannot be excluded that the quality of the studies included in that review may also impact the inconsistent findings of the current work.

## Conclusion

This systematic review showed that inter-professional or multi-professional intervention has no impact on mortality rate but either positive or neutral effects on physical health, psychosocial wellbeing and utilization of health care service. It showed that inter-professional or multi-professional interventions were feasible. It also highlighted the importance of harmonization of assessment tools, to allow direct comparisons for future systematic review.
